# Hydraulic Seal Wear Classification by Fine-Tuning a Transformer-Based Audio Model Using Acoustic Emission

**DOI:** 10.3390/s26092856

**Published:** 2026-05-02

**Authors:** Lisa Maria Svendsen, Vignesh V. Shanbhag, Rune Schlanbusch

**Affiliations:** NORCE Research AS, 5008 Bergen, Norway; vigs@norceresearch.no (V.V.S.); rusc@norceresearch.no (R.S.)

**Keywords:** acoustic emission, seal wear, predictive maintenance, transformer, Audio Spectrogram Transformer, hydraulic cylinder, audio transformer model, audio classification, piston rod seal, bearing strips

## Abstract

Accurate classification of seal wear is essential for condition-based and predictive maintenance of hydraulic cylinders, where seal degradation can cause fluid leakage and impair normal system operation. This study investigates the adaptation of a Transformer-based audio model for classifying seal wear conditions using acoustic emission (AE) signals. Specifically, we adapt the Audio Spectrogram Transformer (AST), a convolution-free, purely attention-based model that operates directly on audio spectrograms. The Transformer architecture enables the modeling of long-range dependencies, while the model learns discriminative representations directly from AE data without relying on manually engineered features. A selective fine-tuning strategy was implemented by adding layer-freezing functionality to the AST training pipeline, enabling different freezing configurations during fine-tuning. This allowed earlier pretrained representations to be preserved while adapting the later layers to the target AE signals, thereby reducing the risk of overfitting in the small-data setting. In addition, validation-driven early stopping was implemented to further improve generalization during fine-tuning. The model was initialized with ImageNet and AudioSet pretrained weights to exploit general-purpose representations learned from large-scale datasets. The AE data were acquired under varying pressure conditions on a hydraulic test rig designed to simulate hydraulic cylinder leakage. The datasets were partitioned into fine-tuning, validation, and evaluation subsets and labeled into three wear states: unworn, semi-worn, and worn. In addition, data augmentation techniques were applied to the fine-tuning data to increase diversity and mitigate class imbalance. The adapted model achieved 97.92% classification accuracy across all wear conditions and pressure settings, demonstrating its ability to learn discriminative wear-related patterns directly from AE data. Furthermore, the framework’s versatility was further assessed on a bearing strip dataset acquired from the same hydraulic test rig. Using the same fine-tuning configuration, the model achieved 95.65% accuracy and 100% recall for the worn state. These findings highlight the potential of transformer-based architectures for data-efficient, end-to-end AE-based diagnostics across hydraulic system components.

## 1. Introduction

Hydraulic cylinders are linear actuators that are used in a wide variety of material handling applications because they provide high pressure and stable speed rate with low energy consumption [1]. However, their operational efficiency and reliability are strongly affected by the condition of the seals. Seal wear can lead to internal and external fluid leakage, resulting in instability of piston rod motion, reduced system efficiency, and ultimately cause unplanned downtime [2,3].

Several studies have applied machine learning techniques to diagnose the seal wear condition and to classify seal wear severity. For example, Kandukuri et al. (2021) [2] utilized acoustic emission (AE) signals with a support vector machine (SVM) to classify unworn, semi-worn, and worn seals, achieving an accuracy of up to 99%. In the study conducted by Noori et al. (2022) [3], a methodology was developed to classify seal wear using AE-based features and an artificial neural network (ANN), also achieving 99% accuracy. In addition, the ANN model was trained and tested directly on filtered raw AE signals without handcrafted feature extraction, where the reported accuracy decreased to approximately 81%. These findings indicate that the high classification performance reported for these SVM- and ANN-based approaches is strongly associated with manually engineered time- and frequency-domain features predefined prior to training. This dependence on manually engineered features can limit scalability and generalization, as the models cannot automatically learn relevant representations from the AE signals.

Within the broader context of applying machine learning, particularly deep learning, to hydraulic cylinder condition monitoring, several studies have explored end-to-end neural network architectures for fault detection. For example, Zhang et al. (2021) [4] combined a Deep Belief Network (DBN) with the CEEMDAN (Complete Ensemble Empirical Mode Decomposition with Adaptive Noise) technique to diagnose internal leakage in hydraulic cylinders using AE signals, achieving an accuracy of approximately 92%. CEEMDAN was used to decompose the AE signals into multiple Intrinsic Mode Functions (IMFs), from which a subset was reconstructed and supplied to the DBN for classification. Although the DBN automatically learned discriminative features from the reconstructed signals, the selection and number of IMFs were manually determined through empirical testing to optimize performance. He et al. (2023) [5] proposed a convolutional neural network (CNN)-based approach to detect internal leakage in hydraulic cylinders of electro-hydrostatic actuators. They aligned multi-source operational signals including pressure, actuator displacement, motor current, and motor rotation speed and applied a feed-forward neural network (FFNN) for data augmentation. A multiscale residual CNN was then trained on the full dataset (original plus augmented data) to extract features, achieving  99% detection accuracy under variable operating conditions. In Guo et al.’s work (2021) [6], a model was proposed to simulate small internal leakage in hydraulic cylinders by converting leakage flow into strain signals using high-precision strain gauges. The collected strain signals were then used to train various neural networks for leakage prediction. The networks included CNN, backpropagation (BP), Takagi–Sugeno (T-S) and Elman where all networks achieved over 90% accuracy, with the CNN showing the best performance. These CEEMDAN-, CNN-, and neural-network-based approaches represent the shift toward deep learning models that learn features directly from data, in contrast to earlier methods that relied on manually engineered AE features combined with shallow classifiers such as SVM or traditional ANN.

Furthermore, within the field of industrial prognostics, recent literature demonstrates that Transformer architectures can outperform traditional CNNs in capturing long-range temporal dependencies in high-frequency acoustic data [7,8]. For example, Ma et al. (2024) [7] proposed a hybrid Transformer network for fault diagnosis in milling tools utilizing AE signals, demonstrating that the attention-based approach outperformed standard convolutional architectures (e.g., CNN, VGG19, and CNN-LSTM) in both noise reduction and feature enhancement. Similarly, Xie and Yang (2024) [8] applied a Swin Transformer to underground AE signals, reporting that the hierarchical Transformer structure surpassed traditional CNNs in both classification accuracy and model convergence speed. However, these recent studies typically rely on complex front-end signal decomposition techniques, such as Wavelets or CELMD (Complex Enhanced Local Mean Decomposition), to preprocess the data. This work therefore extends these efforts by adapting a purely attention-based, end-to-end transformer model for AE-based seal wear classification, which learns discriminative representations directly from the spectrograms. In parallel, following the emergence of deep neural networks, audio classification has transitioned from models based on manually engineered or derived features [9,10] to end-to-end models that directly map audio spectrograms to the corresponding labels [11,12,13,14]. CNNs have been the dominant architecture in end-to-end modeling to learn representations from raw spectrograms [14]. Another approach is the CNN–attention hybrid model, which adds a self-attention mechanism on top of the CNN to capture long-range dependencies.

However, motivated by the success of transformer-based models in the vision domain [15,16,17], Gong et al. (2021) [14] proposed the Audio Spectrogram Transformer (AST). The AST is based on the Transformer architecture [18] and is the first convolution-free and purely attention-based model for audio classification that operates directly on audio spectrograms to effectively capture global context. In addition, to improve AST performance, the authors implemented an approach to transfer knowledge to the AST from the Vision Transformer (ViT) [16], a Transformer-based model for vision tasks pretrained on ImageNet [14]. This cross-modality transfer leverages the similarity between spectrograms and images, addressing the challenge of limited labeled audio data.

Recent work has begun extending AST–based architectures to other industrial condition monitoring tasks. For example, Zhang et al. (2026) [19] proposed a lightweight variant of the AST architecture for pump anomaly detection using acoustic signals, demonstrating accurate fault detection while reducing computational complexity. Their work builds upon the AST framework introduced by Gong et al. (2021) [14] and further demonstrates the suitability of attention-based audio transformers for machinery diagnostics.

In this study, we adapt and fine-tune a pretrained AST model to classify hydraulic seal wear using AE spectrograms. The main contribution of this work lies in adapting a pretrained Transformer-based audio model to classify hydraulic seal wear directly from AE spectrograms without relying on manually engineered features. For the present limited-data setting, the original AST training pipeline was extended with selective layer freezing and validation-driven early stopping to support robust fine-tuning and reduce overfitting. In addition, component-relevant preprocessing was applied to transform the high-frequency AE measurements into inputs compatible with the pretrained model.

By leveraging its attention-based architecture and pretrained weights, the model can learn robust, task-relevant representations directly from AE data without relying on manually engineered features, while mitigating the data-size challenges common in AE research. Furthermore, to assess the adaptability of the AST framework beyond the primary seal-wear task, the model was also fine-tuned and evaluated on a secondary bearing-wear dataset from the same hydraulic test rig. By extending the analysis to bearings, the study broadens the scope beyond a single application case and supports reuse of the adapted AST framework across two AE-based wear-classification tasks.

The remainder of this paper is organized as follows: Section 2 describes the acquisition of the AE data and the architecture of the AST model. Section 3 details the AE preprocessing required for AST compatibility, the data augmentation strategy, and the fine-tuning procedure. Section 4 reports the evaluation results on the held-out dataset. Section 5 discusses the findings within the context of previous studies, outlining the advantages and limitations of the approach, as well as directions for future research. Finally, Section 6 summarizes the main conclusions.

## 2. Methods

### 2.1. AE Data Acquisition

The AE data used to fine-tune, validate and evaluate the AST model in this study were obtained from the tests conducted by (Shanbhag et al., 2020) [20] using a specialized hydraulic test rig, as described in their work and subsequent studies [20,21]. This test rig, comprising an electromechanical cylinder and a hydraulic cylinder head, was designed specifically to simulate internal fluid leakage typical of seal degradation [2]. The operational specifications are summarized in Table 1. To investigate and classify the condition of seal, only the piston rod seal at the top of the cylinder head was replaced [20]. The experiments were conducted under various pressure conditions and seals with three different degrees of wear; unworn, semi-worn and worn. For each seal condition, experiments were performed at four different pressure conditions, 10, 20, 30 and 40 Bar. The three wear states were defined by the physical condition of the seal specimens (unworn, semi-worn, and worn), and leakage severity was assessed by the presence of visible hydraulic fluid (water–glycol) on the piston rod and based on severity of the scratches on the piston rod seal. The AE data were collected using an AE sensor with a frequency range of 50–400 kHz and a resonant frequency of 150 kHz, mounted directly on the piston rod since the piston rod is in direct contact with the piston rod seal. The selection of high-frequency AE sensors over low-frequency vibration monitoring was necessitated by the need for component-specific diagnostics; high-frequency acquisition allows for the separation of seal-related friction noise from the overlapping low-frequency mechanical noise prevalent in complex hydraulic systems [20]. Previous studies (Shanbhag et al., 2020) [20] indicate that seal wear signals are predominantly in the 50–100 kHz range and therefore, all AE signals were bandpass filtered to this range. The sensor was connected through a pre-amplifier (40 dB gain) and a five-meter coaxial cable to the data acquisition system, and testing was performed at a sampling rate of 1 MS/s [2]. The study [20] further reported wear-dependent changes in AE amplitude, separating non-leakage (unworn) from leakage conditions (semi-worn and worn), as well as differences in power spectral density and bandpower within the seal-related frequency band, which enabled discrimination between semi-worn and worn seals. These observations demonstrate that the wear labels correspond to measurable differences in the recorded AE signals [20]. For further details regarding data acquisition and AE signal processing, see [20,21].

Furthermore, the experimental campaign that provided the seal wear data also involved the simultaneous collection of AE data from bearing strips [21]. As detailed in [21], both the piston rod seal and the bearing strips are housed within the same pressurized flange of the test rig. Consequently, the bearing elements were subjected to the same reciprocating motion and pressure conditions as the seals, ranging from 10 to 40 Bar. To accommodate the broader spectral requirements of multi-component monitoring, a wideband AE sensor with an operating range of 15–1000 kHz was utilized for this phase of the study [21]. This additional dataset is utilized in this study as a secondary test case to evaluate the robustness and cross-component generalizability of the adapted AST framework.

### 2.2. The AST Model

**Figure 1:**
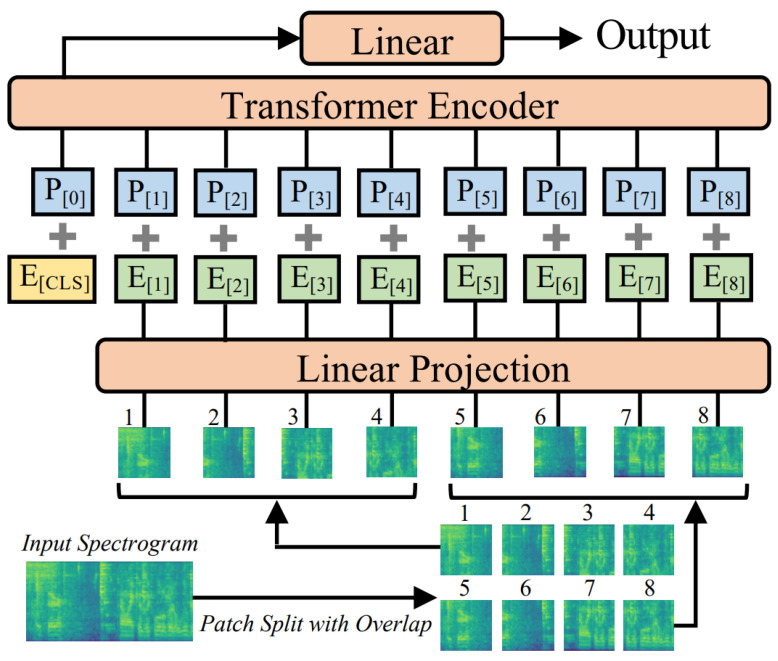
Architecture of the AST. Reproduced from Gong et al. (2021) [14]. The 2D audio spectrogram divided into patches with CLS token prepended.

The architecture: Figure 1 illustrates the AST architecture [14]. Initially, the input audio signal is transformed into a log-Mel spectrogram applying a 25 ms Hanning window with a 10 ms hop size, following Gong et al. (2021) [14]. The 2D spectrogram is then split into a sequence of 16 × 16 patches with an overlap of 6, which are linearly projected into 1D patch embeddings of dimension 768 through a linear projection layer. Each patch embedding is added with a trainable positional embedding, enabling the model to capture the spatial structure of the audio spectrogram. An additional classification token (CLS) is prepended to the sequence, and the resulting sequence is input to a Transformer. To facilitate transfer learning, the AST framework [14] retains the standard Transformer encoder configuration introduced by Vaswani et al. [18] without modification. The Transformer encoder comprises 12 layers and 12 heads with an embedding dimension of 768, consistent with the configurations in [14,17]. The final CLS token is fed to a linear layer, which maps the audio spectrogram representations to labels for classification of the original spectrogram [14]. A key feature of the AST architecture is the Transformer’s self-attention mechanism, which allows each spectrogram patch to interact with all other patches in the input sequence. This enables the model to capture global context across the full spectrogram [14,17].

While Transformer models generally require large-scale datasets for training—and audio datasets are typically smaller than image datasets, the AST architecture overcomes this constraint by leveraging ImageNet pre-training. Since audio spectrograms and images share similar formats, the model can effectively capitalize on cross-modal transfer learning [14,17]. This pretraining not only provides rich, general-purpose representations, but also reduces the typically high data requirements for fine-tuning, which is particularly valuable given the modest size of our AE dataset. For comprehensive details regarding the AST architecture and its official implementation, readers are referred to Gong et al. [14] and the associated public repository [22].

## 3. Fine-Tuning and Adaption of the AST Model

For this study, the AST model was obtained from the official GitHub repository [22] (accessed in January 2025), pretrained on the full AudioSet dataset, more specifically the “Full AudioSet, 10 tstride, 10 fstride, with Weight Averaging (0.459 mAP)” configuration, which represents the best single model reported in the AST paper [14]. Consequently, the model benefits from both ImageNet pretraining via ViT and AudioSet pretraining, providing a strong initialization for downstream audio classification tasks.

Adaptation and fine-tuning of the AST model for hydraulic seal wear classification involved four main stages:

1. Preprocessing of the AE signals:Preparing the signals as required by the AST model.
2. Dataset creation and augmentation: Expanding the fine-tuning dataset to mitigate small size and class imbalance. Creation and labeling of datasets for fine-tuning, validation and evaluation.
3. Implementation of framework extensions: Modifying the training logic to include selective layer-freezing and validation-driven early stopping functionality.
4. Supervised fine-tuning: Fine-tuning the pretrained AST model on the prepared AE dataset with three seal wear classes (unworn, semi-worn, worn).

### 3.1. Preprocessing of AE Data

**Figure 2:**
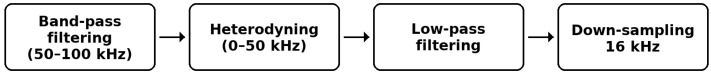
AE signal preprocessing: We cropped the image to reduce the spaces. Please confirm. LMS: Confirmed. pipeline: band-pass filtered (50–100 kHz), heterodyned to 0–50 kHz, low-pass filtered, and downsampled to 16 kHz for AST input.

Before being used for model fine-tuning, the AE signals were preprocessed to ensure compatibility with the input requirements of the AST model. The primary objective of this pipeline is to shift the 50–100 kHz wear signals into a lower frequency range required by the AST model without losing the underlying diagnostic information. The original AE recordings were sampled at 1 MS/s and underwent a sequence of signal processing steps: band-pass filtering, heterodyning, low-pass filtering, and downsampling, as illustrated in the preprocessing pipeline flowchart in Figure 2.

**Figure 3:**
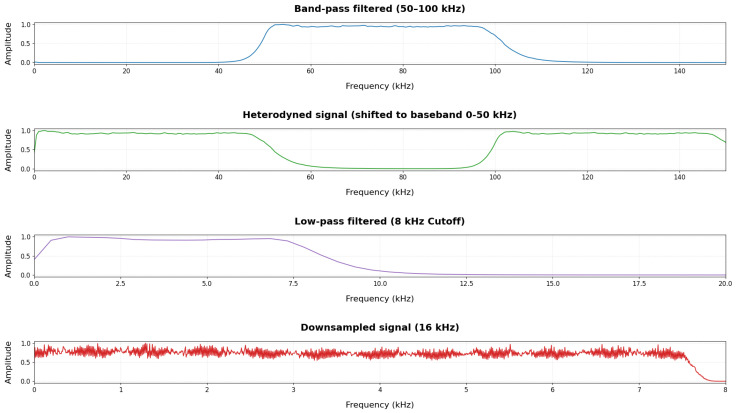
Visualization of the AE signal throughout the preprocessing steps. (1) 50–100 kHz band-pass filtered, (2) Heterodyned signal showing the frequency shift and high-frequency artifacts, (3) Low-pass filtered signal at 8 kHz, (4) Final 16 kHz downsampled signal. All amplitudes are normalized.

1. Band-pass filtering (50–100 kHz): The original AE signals were filtered using a 10th-order Butterworth band-pass filter to isolate the frequency range associated with seal wear and remove irrelevant noise. Previous condition monitoring studies by (Shanbhag et al., 2020) [20] observed that the AE frequencies associated with seal wear fall within this range. This isolated band is shown in Figure 3(1).
2. Heterodyning: To shift the frequency band to a range suitable for audio-based models, the band-passed signal x(t) was heterodyned by multiplication with a 50 kHz cosine local oscillator:
  (1) $$x_{h}\left(t\right)=x\left(t\right)\cos\left(2\pi f_{LO}t\right),\;f_{LO}=50\;\mathrm{kHz}.$$
  This operation preserves the relative spectral structure while moving the 50–100 kHz band down to 0–50 kHz. The resulting frequency shift is illustrated in Figure 3(2).
3. Low-pass filtering: After heterodyning, a 10th-order Butterworth low-pass filter with a cutoff of 50 kHz was applied to remove high-frequency artifacts generated during the heterodyning process. An additional low-pass filter with an 8 kHz cutoff was then applied prior to downsampling to prevent aliasing. The removal of artifacts and isolation of the baseband signal is shown in Figure 3(3).
4. Downsampling: The resulting signal was resampled from 1 MS/s to 16 kHz, producing signals compatible with the AST model which was trained on 16 kHz audio spectrograms. The final preserved signal used for model input can be seen in Figure 3(4).

This preprocessing ensured that each AE sample could be converted into a spectrogram with dimensions consistent with the AST model’s expected input shape, facilitating reliable and efficient feature extraction.

The preprocessing pipeline for the bearing strip dataset followed the same workflow as the seal data: bandpass filtering and heterodyning, followed by downsampling to 16 kHz as required by the AST model. To capture the specific acoustic characteristics of the bearing strips, the bandpass filter was tuned to a 15–30 kHz range, with a corresponding heterodyning frequency of 15 kHz, as these frequencies were found to contain the most significant wear-related information for this component [21]. Aside from these signal-specific frequency adjustments, the pipeline remained identical to maintain evaluation consistency.

### 3.2. Augmentation of AE Data

The original seal dataset contains 252 samples which is a small-sized dataset. In addition, the dataset was imbalanced; there were twice as many unworn samples than worn. To address these limitations, data augmentation was applied exclusively to the fine-tuning data set. The evaluation and validation datasets contain only non-augmented data. The augmentation techniques included Gaussian noise addition, time-stretching, pitch shifting, and temporal shifting, and for each audio file across the classes, multiple augmented versions were generated. These transformations increased the diversity and size of the fine-tuning dataset by approximately 2–2.5 times. Data augmentation is commonly used in machine learning to increase data diversity and reduce the risk of overfitting in data-limited scenarios [23]. However, prior work suggests that augmentation beyond 3–5× the original dataset may produce diminishing returns and introduce artifacts [24]. Therefore, the augmentation factor in this study was limited to approximately 2.5× to increase dataset diversity while avoiding unrealistic signal distortions. The dataset was split into fine-tuning (80%), validation (10%), and evaluation (10%) subsets consistent with the AST model requirements. This resulted in 130 samples per class in the fine-tuning set (390 samples in total), while the validation and evaluation sets remained fixed without augmentation at 16 samples per class (48 samples each) (Table 2). All preprocessed samples, including augmented data, were labeled into the three seal wear classes (unworn, semi-worn, worn) to form the ground-truth dataset for fine-tuning, validation, and evaluation.

### 3.3. Bearing Dataset

For the bearing case study, the dataset comprised a total of 452 samples, which was sufficient for training without the need for synthetic data augmentation. These samples were categorized into three wear states identical to the seal study: unworn, semi-worn, and worn. To maintain methodological consistency, the bearing dataset was partitioned into subsets using the same percentage distribution as the seal study: 80% for fine-tuning, 10% for validation, and 10% for evaluation, resulting in 361, 45, and 46 samples respectively.

To further challenge the model’s robustness, a simple random split was employed for the bearing data without manual class balancing, as shown in Table 3. This approach intentionally introduced a distribution shift between the training and evaluation sets, providing a more rigorous test of the model’s ability to generalize across varying class frequencies without relying on majority-class bias from the training phase. Specifically, while unworn was the majority class in the fine-tuning set (39.9%), semi-worn became the majority class in the evaluation set (45.6%). The high accuracy achieved despite this uncurated distribution suggests that the AST framework learns discriminative acoustic patterns related to wear rather than relying primarily on class-frequency statistics.

### 3.4. Model Adaptation and Fine-Tuning

Modifications were implemented to the original training pipeline focusing on the training loop control logic such as selective layer freezing and early stopping during fine-tuning. The functionality to selectively freeze the AST backbone layers enabled fine-tuning of only the final classification head and a chosen subset of transformer blocks (12 in total). This modification facilitates progressive adaptation to the target dataset while preserving pretrained representations. The early stopping mechanism is based on validation loss and was implemented to prevent overfitting, with a patience parameter specifying the number of consecutive epochs without improvement. Selecting the best model based on validation loss ensures optimal generalization to unseen data [25]. In the present study, training was terminated if the validation loss did not improve for five consecutive epochs, and the checkpoint corresponding to the lowest validation loss was retained for evaluation. Additionally, the training pipeline was modified to save both the model achieving the lowest validation loss (the early-stopped model) and the final model at the end of fine-tuning, allowing post-training evaluation of both checkpoints. Multiple fine-tuning configurations were systematically evaluated, experimenting with hyper-parameters such as the number of unfrozen layers, learning rate, batch size, and augmentation settings among others to identify the configuration that best balanced AE-specific adaptation and generalization. Freezing most layers limited adaptation to the AE domain, while unfreezing all layers led to overfitting, consistent with observations in transformer models for NLP tasks such as BERT and RoBERTa, where partial fine-tuning can achieve near-maximal performance (Lee et al., 2019) [26]. In addition, techniques such as frequency masking, time masking, noise injection, and a learning rate warmup strategy were optionally applied to further enhance model generalization during fine-tuning. The final linear classification head was optimized using Cross Entropy Loss, which directly penalizes incorrect class probability assignments and provides stable gradients for faster convergence (Goodfellow et al., 2016) [27]. The augmented fine-tuning dataset, as described in Section 3.2, was used to fine-tune the model in a supervised manner, with performance evaluated on the validation set after each epoch and validation loss used to monitor fine-tuning progress and trigger early stopping. The final optimal configuration consisted of unfreezing the last five Transformer encoder layers along with the classification head, while the remaining layers retained their pretrained weights, allowing the model to adapt to AE-specific features while preserving general acoustic representations for robust generalization to unseen data.

Table 4 summarizes the hyperparameter values used in the final fine-tuning configuration for both the seal-wear and bearing-wear case.

To evaluate the cross-component generalizability of the framework, the AST model was also applied to a bearing dataset. This test case utilized the identical hyperparameter configuration as the seal study, including the same learning rate, batch size, and layer-freezing strategy (unfreezing the final 5 Transformer layers and the classification head). To test the model’s robustness under uncurated conditions, the bearing dataset was used in its natural, unbalanced state without synthetic augmentation, consisting of 361 fine-tuning samples, 45 validation samples, and 46 evaluation samples (Table 3).

#### Workflow of the Adapted AST Framework for AE-Based Wear Classification

Input: Raw AE signals

Output: Fine-tuned AST model and evaluation metrics

1. Preprocess the raw AE signals using standalone scripts: apply component-relevant band-pass filtering, heterodyning, low-pass filtering, and downsampling to 16 kHz, and save the processed signals as FLAC files.
2. Assign class labels to the processed FLAC files based on filename patterns and construct the dataset JSON file.
3. Split the labeled dataset into fine-tuning, validation, and evaluation subsets using an 80%, 10%, and 10% distribution, respectively.
4. For the seal fine-tuning subset only, apply data augmentation using Gaussian noise addition, time stretching, pitch shifting, and temporal shifting to improve class balance and increase size of the dataset. The bearing fine-tuning subset was used without synthetic augmentation.
5. Set the fine-tuning configuration, including loading the pretrained AST checkpoint, defining the layer-freezing configuration, setting the hyperparameters, and specifying the early-stopping patience.
6. For each fine-tuning epoch:
  (a) Fine-tune the model on the fine-tuning dataset;
  (b) Compute the validation loss ($Loss_{val}$) on the validation subset;
  (c) If $Loss_{val}$ is lower than the best previously recorded validation loss, save the current model weights as the “best” candidate and reset the patience counter to 0;
  (d) If $Loss_{val}$ fails to improve upon the global minimum (the best recorded validation loss so far), increment the patience counter by 1;
  (e) If the patience counter reaches 5, the early-stopping mechanism terminates the fine-tuning process to prevent overfitting.
7. Save the early-stopped model and the final checkpoint model at the end of the fine-tuning.
8. Evaluate both saved models on the held-out evaluation set.
9. Compute accuracy, AUC, evaluation loss, mAP and confusion matrix.

The proposed fine-tuning procedure, including the strategic layer-freezing mechanism and the early stopping criteria, is formally detailed in Algorithm 1. This approach ensures that the high-level features learned from the AudioSet and ImageNet dataset are preserved while the classification head and the final five layers are optimized for specific acoustic emission wear patterns.

### 3.5. Performance Metrics

To evaluate the AST model in classifying wear states, the primary performance metrics considered in this study were accuracy, AUC, and evaluation loss. Since both the seal-wear and bearing-strip tasks are single-label classification problems, accuracy was used as the main evaluation metric, as it directly measures the proportion of correctly classified samples. AUC was included as a supplementary metric to assess class separability, while evaluation loss was monitored to assess agreement between predicted class probabilities and the ground-truth labels. mAP was additionally reported only for completeness and comparability with prior AST-based evaluation practice [14], and was not used as a primary criterion for assessing classification performance.

The accuracy is defined as:

(2) $$\mathrm{Accuracy}=\frac{TP+TN}{TP+TN+FP+FN}.$$

Here, TP, TN, FP, and FN denote the numbers of true positives, true negatives, false positives, and false negatives, respectively. In the present multiclass setting, this corresponds to the ratio of correctly classified samples to the total number of evaluated samples. Accuracy was also reported to maintain consistency with the earlier SVM and ANN-based studies using the same AE data [2,3]. However, accuracy alone may not fully reflect model performance when class boundaries overlap or when some wear states are more difficult to distinguish [28]. Therefore, AUC was additionally included as a supplementary metric to assess how effectively the model separates each class from the others, where an AUC of 1.0 indicates perfect discrimination and an AUC of 0.5 corresponds to random guessing [29]. mAP was also reported as an auxiliary metric to maintain comparability with prior AST-based evaluation practice [14]. In the present study, however, it was included only for completeness and comparability, and was not used as a primary criterion for assessing classification performance. In addition to these scalar performance measures, confusion matrices were examined to provide class-wise insight into the model’s predictions. Unlike overall measures such as accuracy, the confusion matrix reveals which wear states are most frequently confused, thereby enabling a more detailed interpretation of model behavior [30]. Finally, the evaluation loss was monitored to assess model behavior during evaluation. A lower loss indicates better agreement between the predicted class probabilities and the ground-truth labels, thereby providing an additional indication of model convergence and prediction confidence.
**Algorithm 1** AST Fine-Tuning with Selective Layer Unfreezing and Early Stopping**Require:** Pre-trained AST weights Θ, Fine-tuning dataset $D_{fine}$, Validation dataset $D_{val}$, Evaluation set $D_{eval}$, Patience P=5, Max Epochs $N_{max}=30$**Ensure:** Optimal Model $\Theta^{*}$ (Early-stopped), Final Epoch Model $\Theta_{final}$1:**1. Model Initialization & Freezing Mechanism:**2:Set requires_grad←False for all parameters in Θ    ▹ Freeze all layers3:Unfreeze classification head and final 5 Transformer layers ($B_{8\ldots 12}$)4:Initialize $Loss_{best}\leftarrow \infty$, counter←05:**2. Fine-Tuning and Early Stopping Loop:**6:**for** each $epoch=1,2,\ldots ,N_{max}$ **do**7:    $\Theta \leftarrow \mathrm{Train}(\Theta ,D_{fine})$     ▹ Update only 5 layers + classification head8:    $Loss_{val}\leftarrow \mathrm{Validate}\left(\Theta ,D_{val}\right)$9:    **if** $Loss_{val}<Loss_{best}$ **then**          ▹ New global minimum found10:        $Loss_{best}\leftarrow Loss_{val}$11:        $\Theta^{*}\leftarrow \Theta$          ▹ Save early-stopped model checkpoint12:        counter←0                ▹ Reset patience tracker13:    **else**14:        counter←counter+115:        **if** counter≥P **then**             ▹ Patience limit reached16:           $\Theta_{final}\leftarrow \Theta$         ▹ Save state at fine-tuning termination17:           **break**                ▹ Early stopping triggered18:        **end if**19:    **end if**20:**end for**21:**3. Model Evaluation:**22:$M^{*}\leftarrow \mathrm{Evaluate}\left(\Theta^{*},D_{eval}\right)$        ▹ Evaluate Early-stopped Model23:$M_{final}\leftarrow \mathrm{Evaluate}\left(\Theta_{final},D_{eval}\right)$       ▹ Evaluate Final Epoch Model24:**return**$\Theta^{*}$, $\Theta_{final}$, and Metrics $\left\{M^{*},M_{final}\right\}$

## 4. Results

### 4.1. Evaluation of Seal Wear

The performance of the fine-tuned AST model was evaluated on a held-out dataset that was not used during fine-tuning. This dataset contained 16 samples per class (48 samples total), providing balanced representation across the three classes. Evaluation was performed on the checkpoint achieving the lowest validation loss (early-stopped model) and the final checkpoint saved at the end of fine-tuning.

The early-stopped model achieved high performance with an overall accuracy of 97.92%, an AUC of 0.9954, and an evaluation loss of 0.121 (Table 5). These results indicate strong classification performance, excellent class separability, and good agreement between the predicted probabilities and the ground-truth labels. In addition, a high mAP value of 0.9925 was observed, which is consistent with the overall performance and is reported for completeness and comparability with prior AST-based evaluation practice [14]. By contrast, the final checkpoint at the end of fine-tuning achieved slightly lower accuracy (93.75%), highlighting the benefit of early stopping in identifying the model checkpoint with the best generalization.

**Figure 4:**
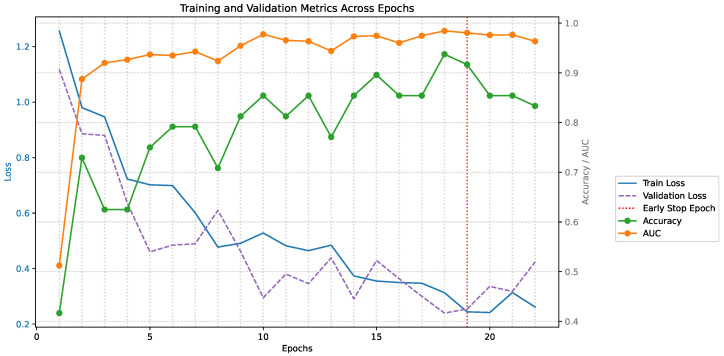
Fine-tuning and validation loss, and accuracy and AUC over epochs during fine-tuning of the AST model.

Figure 4 shows the fine-tuning and validation loss over the course of fine-tuning the AST model. Early stopping was triggered at epoch 19, with the minimum validation loss occurring at epoch 18. Both the early-stopped model (epoch 18) and the final model (epoch 23) were saved, allowing post-training evaluation of both checkpoints. Layer freezing was applied to the backbone to preserve pretrained representations while fine-tuning the classification head and selected transformer blocks.

**Figure 5:**
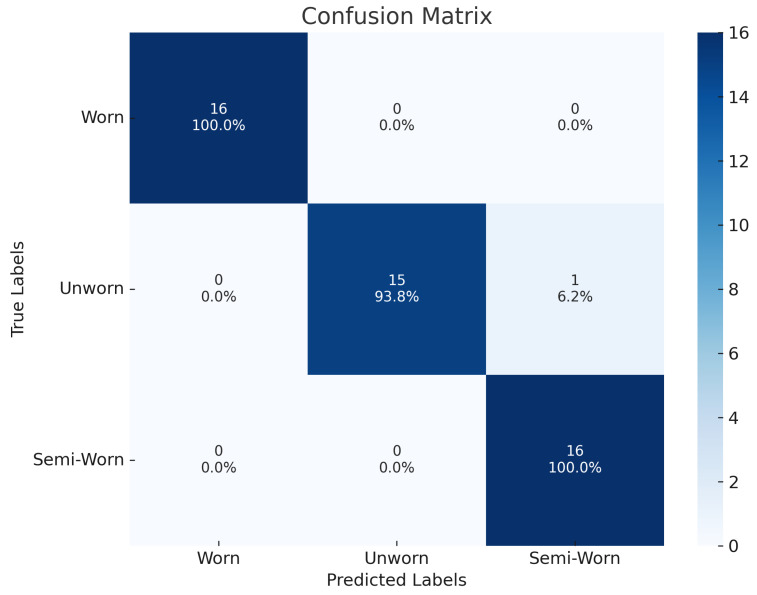
Confusion matrix from the evaluation of seal of the fine-tuned model.

Class-wise analysis using a confusion matrix, Figure 5, revealed only one misclassification, where the model predicted the seal as semi-worn instead of unworn, demonstrating strong discriminative ability across all classes.

These results suggest that the fine-tuned AST model robustly classifies seal wear conditions with minimal error on unseen AE data and that early-stopping effectively identifies the model checkpoint with optimal generalization.

Despite the modest size of the dataset, the model demonstrated strong adaptability, achieving stable and accurate performance through careful hyperparameter tuning. Consistent with previous studies [31,32,33], a relatively low learning rate was employed to stabilize fine-tuning, mitigate overfitting, and ensure effective learning from limited data. Prior research [31,33] indicates that smaller datasets particularly benefit from lower learning rates, promoting smooth convergence and improved generalization.

### 4.2. Evaluation of Bearing Wear

To evaluate the framework’s versatility across different mechanical components, the adapted AST model was fine-tuned and evaluated on the bearing strip dataset described in Section 3.3. To ensure a rigorous assessment of the architecture’s robustness, the fine-tuning process utilized the same hyperparameters and training configuration as the seal wear classification task (Table 4). Under this identical configuration, including unfreezing the final five Transformer layers, the early stopping was triggered at epoch 11. Following the specified patience of five epochs, the fine-tuning process was terminated at epoch 15. A comparison between the checkpoints reveals that the early-stopped model (epoch 10) achieved an overall accuracy of 95.65%, while the final checkpoint model (epoch 15) resulted in a lower accuracy of 93.48%. This divergence, summarized in Table 6, supports the use of validation-loss-driven early stopping to identify the point of optimal generalization and prevent the degradation in performance observed in the final stages of training.

This result suggests that the adapted AST framework can generalize to the acoustic signatures of different mechanical components, extending from seals to bearing strips without requiring manually engineered features or task-specific changes to the core architecture.

**Figure 6:**
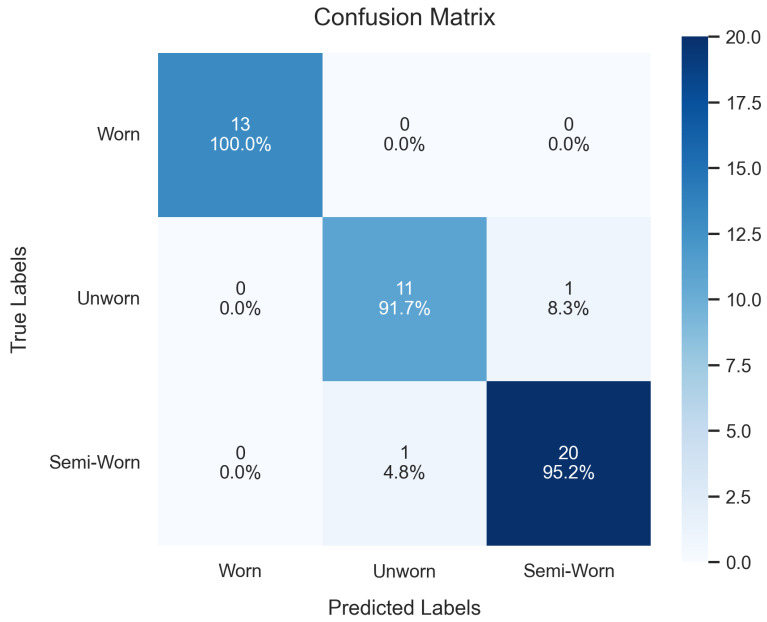
Confusion matrix from the evaluation of the bearing of the fine-tuned model.

Class-wise analysis using a confusion matrix, Figure 6, revealed two misclassifications: one where the model predicted semi-worn instead of unworn, and another where it predicted unworn instead of semi-worn. This indicates strong discriminative ability across all classes, particularly with the 100% accuracy achieved for the worn state.

The high accuracy maintained across different mechanical components and distinct frequency bands suggests that the AST framework can learn transferable acoustic patterns associated with degradation.

## 5. Discussion

The present study serves as an initial investigation of transformer-based representation learning for AE-based seal wear classification using an established experimental dataset previously analyzed with SVM and ANN approaches [2,3]. This work should therefore be interpreted as part of a methodological progression using the same experimental AE dataset, enabling comparison within a consistent experimental framework. As shown in Table 7, when compared with these earlier methods, which relied on manually engineered AE features, the adapted AST model achieves comparable classification accuracy while learning discriminative representations directly from spectrogram inputs. Notably, while the baseline ANN achieved high accuracy with manually engineered features, its performance dropped to 81% when trained on filtered raw AE signals without such prior feature selection. Consequently, this study demonstrates the feasibility of attention-based representation learning for AE-based seal wear diagnostics.

Observations reported in the original experimental studies [20,21] showed that increasing seal wear produces measurable changes in AE amplitude and spectral characteristics reflected by power spectral density and bandpower within the seal-related frequency range. Since the wear labels correspond to physically defined seal conditions and measurable AE signal differences, the discriminative patterns learned by the AST model can be interpreted as wear-related features learned directly from the AE data. This interpretation can be linked to the Transformer’s self-attention mechanism, which enables the AST to model relations across spectrogram patches and thereby learn wear-related time-frequency patterns directly from the AE representation without requiring manually engineered features.

To evaluate performance and adaptability of the AST framework beyond the primary seal classification task, a secondary test case was performed using a bearing strip dataset. As summarized in Table 8, the high classification accuracy (95.65%) achieved on this secondary component supports the adaptability of the framework across different mechanical components and acoustic frequency ranges. Notably, this performance was maintained on a non-curated dataset where the class distribution in the evaluation set differed from the fine-tuning set. While the model was primarily exposed to unworn samples during training, it correctly identified semi-worn as the majority class during evaluation without loss in predictive capability. This suggests that the Transformer’s attention mechanism can capture discriminative acoustic patterns related to degradation rather than simply learning the class-frequency statistics of the fine-tuning data. Furthermore, the 100% recall for the worn state is of particular industrial significance, as the failure to detect a worn bearing typically carries much higher maintenance costs and safety risks than a false alarm [34].

A detailed side-by-side comparison of the model’s performance and convergence behavior across both mechanical components is provided in Table 8.

Transfer learning played an important role in enabling effective model training with limited AE data. The pretrained AST model leverages representations learned from large-scale visual (ImageNet) and audio (AudioSet) datasets, facilitating stable convergence during fine-tuning and reducing the amount of labeled AE data required. In addition, only the final five transformer layers and the classification head were unfrozen during fine-tuning, while the earlier layers remained frozen. This selective fine-tuning strategy represents a parameter-efficient adaptation of the AST, which is critical for mitigating overfitting in data-constrained industrial environments. This approach allowed the model to retain general acoustic feature representations learned during pretraining while adapting higher-level representations to the specific characteristics of AE signals generated by hydraulic seal wear.

Furthermore, the high classification accuracy achieved despite the niche, low-volume nature of the experimental AE datasets suggest that the adapted transformer-based framework can learn effectively from limited labeled data, indicating its suitability for industrial AE-based diagnostics even when large-scale fault datasets are unavailable. Additionally, it can be observed by comparing the result from the evaluation of both the early-stopped model and the final model, that validation-driven early stopping helps maintain classification accuracy on unseen data.

While the current results demonstrate high data efficiency, further validation on larger and independent experimental datasets would strengthen confidence in the generalizability of the proposed approach. Future work should therefore prioritize the acquisition of additional AE measurements to increase dataset size, improve class balance, and enable validation across independent datasets collected under different operating conditions. Morever, systematic benchmarking against alternative transformer-based audio architectures and the integration of explainable artificial intelligence (XAI) techniques could provide deeper insight into the spectro-temporal features used for classification, improving interpretability and supporting the physical understanding of wear-related AE patterns. In addition, future work could also include the integration of this framework into real-time edge computing devices, leveraging the model’s efficient convergence and selective fine-tuning strategy for continuous, autonomous health monitoring in industrial environments.

## 6. Conclusions

To the best of our knowledge, transformer-based audio architectures have not previously been applied to AE-based seal wear classification in hydraulic cylinders. Overall, the contribution of this study is to show that a pretrained AST framework can be effectively adapted to AE-based wear classification under limited-data conditions by implementing controlled layer freezing during fine-tuning, validation-driven early stopping, and signal preprocessing to convert the high-frequency AE measurements into model-compatible inputs. In summary, the adapted AST framework demonstrated accurate classification on unseen AE seal data, achieving an accuracy of 97.92% for the seal wear, successfully distinguishing between unworn, semi-worn, and worn seal conditions. The model automatically learned discriminative representations from preprocessed AE spectrograms, eliminating the need for manually engineered features and capturing patterns intrinsically associated with seal wear. Furthermore, by using a model pretrained on large-scale datasets and implementing a selective fine-tuning strategy with controlled layer freezing, the framework adapted effectively to the specific characteristics of AE signals while mitigating overfitting in the limited-data setting. These results highlight the potential of purely attention-based transformer architectures for AE-based seal-wear diagnostics in condition-monitoring applications.

The framework was also applied to an additional bearing strip dataset from the same hydraulic test rig. Using the same fine-tuning configuration, the adapted AST model achieved 95.65% accuracy and 100% recall for the worn state, indicating that the framework can be reused across two AE-based wear-classification tasks within the same experimental platform.

## Figures and Tables

**Table 1:** The operational specification of the hydraulic test rig. Reproduced from Shanbhag et al. (2020) and Shanbhag et al. (2021) [20,21].

Parameter	Value
Setup	Hydraulic test rig
Number of sensors	1
Seal material	Polyether-based polyurethane elastomer
Coating on piston rod	Cladded coating of a cobalt-based alloy
Bearings	Orkot Slydring (C380 grade)
Fluid	Water glycol
Speed	100 mm/s
Pressure	10, 20, 30, 40 Bar
Stroke length	600 mm
Number of strokes	5
AE data acquisition	1 MS/s
AE amplifier gain	40 dB
Seal condition	Unworn, Semi-worn, Worn
Bearing condition	Unworn, Semi-worn, Worn

**Table 2:** Datasetpartition for the seal-wear case study.

Parameter	Value
Fine-tuning set	130 samples per class (390 total)
Validation set	16 samples per class (48 total)
Evaluation set	16 samples per class (48 total)

**Table 3:** Class distribution across the bearing dataset partitions, showing the natural distribution shift resulting from a simple random split.

Dataset Partition	Worn	Unworn	Semi-Worn	Total
Total Dataset	112 (24.8%)	175 (38.7%)	165 (36.5%)	452
Fine-tuning (80%)	88 (24.4%)	144 (39.9%)	129 (35.7%)	361
Validation (10%)	11 (24.4%)	19 (42.2%)	15 (33.3%)	45
Evaluation (10%)	13 (28.3%)	12 (26.1%)	21 (45.6%)	46

**Table 4:** Fine-tuning hyperparameters configuration for the adaptedAST model.

Hyperparameter	Value
Optimizer	Adam
Weight decay	0.01
Batch size	8
Epochs	30
Mixup rate	0.2
Frequency Masking	Max 56 bins
Time Masking	Max 350 frames
Learning Rate	0.0001
Audio frames	1300 per sample
Dataset mean	−4.27
Dataset std	4.57
Total parameters	87.99 million
Trainable parameters	35.44 million
Main metric	Accuracy
Loss function	CrossEntropyLoss
Number of workers	2
Noise augmentation	True
Balanced sampling	None
Frequency stride	10
Time stride	10
Weight averaging	False
Warmup strategy	True
Using ImageNet pre-training	True
Using AudioSet pre-training	True
Early stopping	Based on validation loss
Early stopping patience	5
Unfrozen layers	Last 5 Transformer encoder layers + classification head
LR scheduler	MultiStepLR starting at epoch 6, decay rate 0.8 every 4 epochs

**Table 5:** Evaluation metrics for the AST model on the held-out AE dataset. Results are shown for the early-stopped model and the final checkpoint model.

Metric	Early-Stopped Model	Final Checkpoint Model
Overall Accuracy	97.9167%	93.75%
Area Under ROC Curve (AUC)	0.995443	0.995443
Evaluation Loss	0.120544	0.144165
Mean Average Precision (mAP)	0.992512	0.992080

**Table 6:** Evaluation metrics for the AST model on the held-out bearing AE dataset. Results are shown for the early-stopped model and the final checkpoint model.

Metric	Early-Stopped Model	Final Checkpoint Model
Overall Accuracy	95.6522%	93.4783%
Area Under ROC Curve (AUC)	0.992740	0.993287
Evaluation Loss	0.192993	0.147583
Mean Average Precision (mAP)	0.984880	0.991653

**Table 7:** Comparative performance of AST against SVM and ANN baselines using the same AE dataset.

Model	Architecture	Input Processing	Accuracy (%)
SVM [2]	SVM	16 Manually Engineered Features	99.00%
ANN (Case 1) [3]	Shallow ANN	16 Manually Engineered Features	99.30%
ANN (Case 2) [3]	Shallow ANN	Filtered Raw AE Signals	81.00%
AST (Ours)	Transformer	Log-Mel Spectrogram	97.92%

**Table 8:** Cross-component performance comparison of the AST framework.

Metric	Piston Rod Seal	Bearing Strip
Frequency Band	50–100 kHz	15–30 kHz
Min. Validation Loss (Epoch)	18	10
Termination (Epoch)	23	15
Overall Accuracy	97.92%	95.65%
Recall (Worn State)	100%	100%

## Data Availability

The AE data used in this study were obtained from experiments originally conducted by (Shanbhag et al., 2020) [20] and reused with appropriate citation. No new raw AE data were generated in this study. Processed data and trained model checkpoints may be made available from the corresponding author upon reasonable request.
